# Association between untreated and treated blood pressure levels and cognitive decline in community-dwelling middle-aged and older adults in China: a longitudinal study

**DOI:** 10.1186/s13195-024-01467-y

**Published:** 2024-05-10

**Authors:** Haibin Li, Man Wang, Frank Qian, Zhiyuan Wu, Weida Liu, Anxin Wang, Xiuhua Guo

**Affiliations:** 1 Department of Cardiac Surgery, Beijing Chaoyang Hospital, Capital Medical University, No. 8 Gongren Tiyuchang Nanlu, Chaoyang District, 100020 Beijing, China; 2 Heart Center and Beijing Key Laboratory of Hypertension, Beijing Chaoyang Hospital, Capital Medical University, Beijing, China; 3 Beijing Municipal Key Laboratory of Clinical Epidemiology, Beijing, China; 4 Department of Cardiology, Cardiovascular Center, Beijing Friendship Hospital, Capital Medical University, Beijing, China; 5 Section of Cardiovascular Medicine, Boston Medical Center, Boston University Chobanian & Avedisian School of Medicine, Boston, MA USA; 6 Department of Nutrition, Harvard T.H. Chan School of Public Health, Boston, MA USA; 7 State Key Laboratory for Complex, Severe, and Rare Diseases, Peking Union Medical College Hospital, Beijing, China; 8 Department of Neurology, Beijing Tiantan Hospital, Capital Medical University, Beijing, China; 9 Beijing Tiantan Hospital, China National Clinical Research Center for Neurological Diseases, Capital Medical University, Beijing, China; 10 Department of Epidemiology and Health Statistics, School of Public Health, Capital Medical University, Beijing, China

**Keywords:** Blood pressure, Cognitive decline, Population‑based surveys, Longitudinal analysis

## Abstract

**Background:**

Optimal blood pressure (BP) levels to reduce the long-term risk of cognitive decline remains controversial. We aimed to investigate the association between BP and anti-hypertensive treatment status with cognitive decline in older adults.

**Methods:**

This study used data from the China Health and Retirement Longitudinal Study. Cognitive function was assessed at year 2011, 2013, 2015, and 2018. Global cognitive Z-score was calculated as the average score of episodic memory and mental intactness. BP were measured at the first and second wave. Pulse pressure (PP) was calculated as systolic BP (SBP) minus diastolic BP. Cumulative BP was calculated as the area under the curve using BP measurements from 2011 to 2013. Linear mixed models were used to assess the longitudinal association between BP-related measurements and cognitive decline.

**Results:**

We included 11,671 participants (47.3% men and mean age 58.6 years). Individual with BP > 140/90 mm Hg or taking anti-hypertensive medication were independently associated with accelerated cognitive decline (*β*=-0.014, 95% CI: -0.020 to -0.007). Individuals with anti-hypertensive medication use, but with controlled SBP to less than 120 mm Hg did not have a significantly increased risk of cognitive decline compared with normotension (*β*=-0.003, 95% CI: -0.021 to 0.014). Individuals on anti-hypertensive treatment with PP of more than 70 mm Hg had a significantly higher risk of cognitive decline (*β*=-0.033, 95% CI: -0.045 to -0.020). Regardless of anti-hypertensive treatment status, both elevated baseline and cumulative SBP and PP were found to be independently associated with accelerated cognitive decline.

**Conclusions:**

Cumulatively elevated SBP, PP and uncontrolled BP were associated with subsequent cognitive decline. Effectively controlling BP with anti-hypertensive treatment may be able to preserve cognitive decline in older adults.

**Supplementary Information:**

The online version contains supplementary material available at 10.1186/s13195-024-01467-y.

## Introduction

Dementia is a prevalent health condition in later life that significantly affects a substantial proportion of the global population [1]. According to the Global Burden of Diseases, Injuries, and Risk Factors Study (GBD) 2019, there were 57.4 million people worldwide living with dementia in 2019, and the number is projected to increase to 152.8 million by the year 2050 [1]. To date, there have been no proven interventions that effectively prevent or delay the incidence of dementia or cognitive decline.

The 2020 report of *Lancet* Commission on Dementia Prevention, Intervention, and Care shows that midlife hypertension is one of most important modifiable risk factors for preventing dementia [2]. Furthermore, a recent systematic review and meta-analysis of 209 prospective studies also demonstrated that exposure to high blood pressure (BP) were significantly associated with increased risk of dementia and cognitive impairment [3]. However, results from the Systolic Blood Pressure Intervention Trial (SPRINT) showed that intensive BP control did not significantly reduce the risk of probable dementia among adults with hypertension [4]. Results from observational studies have been mixed, with some demonstrating a significant association between elevated BP and risk of cognitive decline [5–11], while other studies have not [12–14]. One possible explanation for the inconsistent findings is the variation in cognitive assessment methods, differences in follow-up duration, and varying characteristics of the study population. Moreover, it is important to note that most of these studies were conducted exclusively in Western populations [5–11]. In turn, the relationship between BP levels and cognitive decline among Chinese population remain less well-characterized [15, 16], despite often having a higher prevalence of hypertension (particularly undiagnosed and/or undertreated hypertension) and cerebrovascular disease [15, 16].

In addition to the study of single BP measurement at baseline, several studies have used repeated BP measurements to examine the association of cumulative BP exposure with midlife cognitive function, cognitive decline, and dementia, with mixed findings [17–19]. It remains uncertain whether cumulative BP is associated with a faster rate of cognitive decline in a general population of Chinese middle-aged and older adults.

Therefore, we aimed to investigate the longitudinal associations between baseline BP level and hypertension treatment status with subsequent cognitive decline over the span of 7 years, using data from a large, nationally representative cohort of Chinese middle-aged and older adults. We additionally evaluated the relationship between cumulative BP exposure and cognitive decline.

## Methods

### Study design and participants

The China Health and Retirement Longitudinal Study (CHARLS) began in 2011 (first phase of data collection referred to as wave 1) and is an ongoing national survey of a representative sample of Chinese residents aged 45 years and older [20]. Briefly, a total of 17,708 individuals from 10,257 households were collected using a multistage stratified probability-proportionate-to-size sampling method. The CHARLS study covered 150 counties/districts, and 450 villages or urban communities across 28 provinces in China. Follow-up was conducted at intervals of two or three years, comprising wave 2 (2013), wave 3 (2015), and wave 4 (2018). Data on demographic characteristics, medical history, prescription drug usage, and cognitive function were collected. All participants provided informed consent. CHARLS were approved by the Biomedical Ethics Committee of Peking University (IRB00001052-11015).

**Fig. 1:**
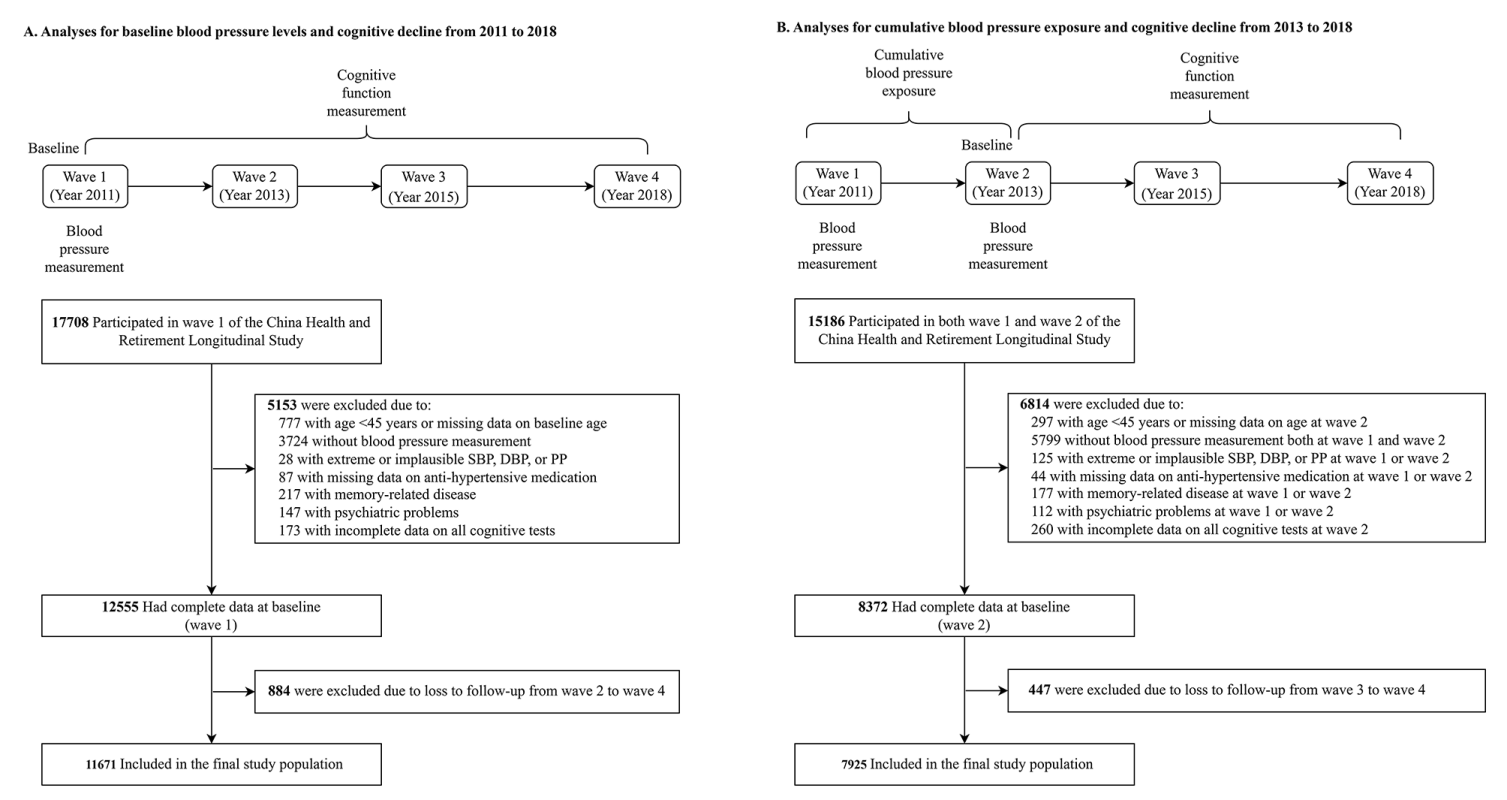
Flowchart of participants

For the current analyses, we utilized 7 years of data spanning from wave 1 (2011) to wave 4 (2018) to investigate the association between baseline BP levels and cognitive decline. Additionally, we utilized data from wave 2 (2013) to wave 4 (2018) to investigate the relationship between cumulative BP exposure and cognitive decline. The study timeline and flow chart of the analytical sample are presented in Fig. 1. Participants were excluded if they met any of the following criteria: (1) age < 45 years or had missing data on baseline age; (2) did not have BP measurement; (3) had extreme or implausible BP values (i.e., systolic BP [SBP] < 80 or > 250 mm Hg; diastolic BP [DBP] < 40 or > 150 mm Hg; pulse pressure [PP, defined as the difference between SBP and DBP] ≤ 15 mm Hg); (4) had missing data on anti-hypertensive medication; (5) had a documented history of neurocognitive disorders (i.e., dementia and/or Parkinson’s disease); (6) had a history of psychiatric diagnoses; (7) had incomplete cognitive measurements at baseline; and (8) had no cognitive measurements during follow-up. Finally, a total of 11,671 and 7,925 participants were included in the analysis of baseline BP levels and cumulative BP exposure with cognitive decline, respectively.

### Measurements of BP

During the physical examination in CHARLS, trained staff measured BP three times at 45-second intervals using Omron digital devices (Omron™ HEM-7200 Monitor) [20]. The average of the second and third BP measurements was used for the analysis. We categorized the study participants into four groups: (1) SBP < 120 and DBP < 80 mm Hg (i.e., normal BP); (2) SBP of 120–129 and DBP < 80 mm Hg (i.e., elevated BP); (3) SBP of 130–139 or DBP of 80–89 mm Hg (i.e. stage I hypertension); (4) people with SBP ≥ 140 or DBP ≥ 90 mm Hg (i.e. stage II hypertension) or taking anti-hypertensive medication according to the 2017 American College of Cardiology (ACC)/American Heart Association (AHA) hypertension guidelines [21]. Respondents were classified as currently taking anti-hypertensive medication if they answered “yes” to the question “Are you now taking any of the following treatments to treat or control your hypertension?”. As per a previous study [22], SBP was classified into four groups: <120, 120–129, 130–139, and ≥ 140 mm Hg; DBP was classified into three groups: <80, 80–89, and ≥ 90 mm Hg; PP was classified into four groups: <50, 50–59, 60–69, and ≥ 70 mm Hg.

### Calculation of cumulative BP exposure

BP measurements (SBP, DBP, and PP) from 2 visits (wave 1 and wave 2) were used to analyze the cumulative exposure. According to previous studies [17, 19, 23], we used area under the curve over 2 years to estimate cumulative BP exposure (mm Hg × years) for each participant, which was calculated by connecting all points in a direct line using the composite trapezoid rule. The cumulative BP exposure was subsequently categorized into three groups: low (bottom tertile), medium (middle tertile), and high (top tertile).

### Measurements of cognitive function

At each wave of CHARLS, participants underwent a battery of 2 cognitive tests, which have been previously published [24, 25]. First, mental intactness was assessed based on components of the Telephone Interview of Cognitive Status (TICS) battery, which included performing serial 7 subtraction from 100 (up to five times), naming today’s date (month, day, year, and season), and testing the ability to redraw a picture that was shown to the respondents. The mental intactness score ranged from 0 to 10. Second, episodic memory was assessed by immediate and delayed word recall tasks. Immediate and delayed recall scores ranged from 0 to 10, and a composite memory score was created by averaging the scores of the 2 individual memory tests.

To enable direct comparisons across cognitive tests, Z scores were calculated for each cognitive score according to the baseline score by subtracting the mean and dividing by the SD. The Z score for global cognitive function was calculated by averaging the Z scores for the 2 tests and re-standardizing to baseline according to the mean and SD of the baseline global cognitive Z scores, greater indicates better cognition, in alignment with prior study [26].

### Covariates

Baseline covariates information were collected by face-to-face questionnaire and included sociodemographic characteristics (age, sex, marital status [married or not married], education [< middle school, middle school, or ≥ high school], area of residence [urban or rural], and health insurance [yes or no]), lifestyle factors (smoking and alcohol status [never, former, current]), self-reported medical history (cardiovascular diseases [heart diseases or stroke], diabetes, dyslipidemia, kidney disease, lung disease, and cancer), self-reported medication use (hypoglycemic medication, lipid-lowering medication, and anti-hypertensive medication), body-mass index (BMI), and depressive symptoms. Elevated depressive symptoms were defined as a score ≥ 10 on the 10-item Centers for Epidemiologic Studies of Depression Scale (CES-D-10) [27].

### Statistical analyses

Continuous variables were described using mean ± SD. Categorical variables were described using frequencies and proportions. Differences in characteristics were compared using one-way analysis of variance or Pearson’s *χ*^2^ test as appropriate.

First, to examine the relationship between baseline BP and cognitive decline, we used linear mixed-effects models with random intercepts and slopes to assess the association between BP categories, elevated SBP, DBP and PP, and by whether or not individuals were taking anti-hypertensive medication status, and cognitive decline over study follow-up. Unstructured covariance structure was used to account for repeated cognitive measurements. The longitudinal association between BP measures and cognitive decline was evaluated by the interaction of the BP metrics and the follow-up time (i.e., SBP/DBP categories × time). Model 1 was adjusted for baseline age, age-squared, and sex. Model 2 was further adjusted for education, marital status, residence, health insurance, smoking status, drinking status, history of cardiovascular diseases, diabetes, dyslipidemia, kidney disease, lung disease, and cancer, depressive symptoms, hypoglycemic medication use, lipid-lowering medication use, BMI, and BMI-squared.

Second, we investigated the potential dose-response relationship between continuous SBP, DBP, PP and cognitive decline. We first incorporated linear and quadratic terms for SBP, and their interaction with time into linear mixed-effects models (model 2), with additional adjustments for anti-hypertensive medication status at baseline. We then calculated the rate of cognitive decline using the Stata *margins* command. Similar analyses were conducted for DBP and PP.

Third, we used linear mixed-effects models to examine the association between cumulative BP metrics during wave 1 to wave 2 and subsequent cognitive decline during wave 2 to wave 4. We adjusted for covariates in model 2 plus time-updated anti-hypertensive medication status from wave 1 to wave 2.

Examine the robustness of our results, we performed several subgroup and sensitivity analyses. First, we conducted subgroup analyses by baseline age (< 60 or ≥ 60 years) and sex. Second, we repeated our analysis after excluding those with cardiovascular diseases, diabetes, or both at baseline. Third, we repeated the dose-response relationship between BP level and cognitive decline stratified by the use of anti-hypertensive medication status. Finally, to test the robustness of dose-response relationship between BP and cognitive decline, we repeated the main analyses with additional categories of SBP (< 120, 120–129, 130–139, 140–149, 150–159, 160–169, and ≥ 170 mm Hg), DBP (< 60, 60–69, 70–79, 80–89, and ≥ 90 mm Hg), and PP (< 40, 40–49, 50–59, 60–69, and ≥ 70 mm Hg).

All statistical analyses were conducted using Stata version 17.0 (StataCorp), with a two-tailed *P* value < 0.05 considered to be statistically significant, unless otherwise stated.

## Results

### Study population

At baseline, among the 11,671 participants included, the mean ± SD age of the participants was 58.6 ± 9.0 years, 5,516 participants (47.3%) were male, and 7,465 (64.0%) lived in rural areas. The prevalence of those with SBP/DBP < 120/<80 mm Hg was 33.6% (3,901 of 11,671), and the prevalence of those with SBP/DBP ≥ 140/90 mm Hg or taking anti-hypertensive medication use was 37.1% (4,300 of 11,671; Table 1). Compared with individuals with SBP/DBP < 120/80 mm Hg, those with SBP/DBP ≥ 140/90 mm Hg or taking anti-hypertensive medication were more likely to be female, older, have lower education levels and greater number of comorbid conditions (Table 1).

**Table 1:** Baseline characteristics of participants in the china health and retirement longitudinal study

	Blood Pressure Categories				
	< 120/<80 mm Hg (*n* = 3901)	120–129/<80 mm Hg (*n* = 1444)	130–139/80–89 mm Hg (*n* = 1947)	≥ 140/90 mm Hg or taking anti-HTN (*n* = 4300)	*P* value
Age, y	56.1 ± 8.1	58.4 ± 8.8	57.6 ± 8.7	61.2 ± 9.3	< 0.001
Men	1759 (45.1)	714 (49.4)	1038 (53.3)	1959 (45.6)	< 0.001
Married	3400 (87.2)	1212 (83.9)	1634 (83.9)	3461 (80.5)	< 0.001
Educational level					< 0.001
< Middle school	2621 (67.2)	956 (66.2)	1279 (65.7)	3093 (71.9)	
Middle school	838 (21.5)	333 (23.1)	415 (21.3)	792 (18.4)	
≥ High school	442 (11.3)	155 (10.7)	253 (13.0)	415 (9.7)	
Rural residence	2678 (68.6)	931 (64.5)	1239 (63.6)	2567 (59.7)	< 0.001
Health insurance, yes	3653 (93.9)	1365 (94.9)	1813 (93.4)	4018 (93.7)	0.31
Smoking status					< 0.001
Never	2427 (62.2)	847 (58.7)	1115 (57.3)	2641 (61.4)	
Former	272 (7.0)	119 (8.2)	171 (8.8)	421 (9.8)	
Current	1202 (30.8)	478 (33.1)	661 (33.9)	1237 (28.8)	
Alcohol status					< 0.001
Never	2360 (60.5)	855 (59.2)	1069 (54.9)	2550 (59.3)	
Former	285 (7.3)	86 (6.0)	134 (6.9)	414 (9.6)	
Current	1254 (32.2)	503 (34.8)	743 (38.2)	1335 (31.1)	
Medical history, yes					
Cardiovascular diseases	342 (8.8)	130 (9.0)	200 (10.3)	841 (19.6)	< 0.001
Diabetes	135 (3.5)	59 (4.1)	95 (4.9)	400 (9.4)	< 0.001
Dyslipidemia	212 (5.5)	90 (6.4)	129 (6.8)	659 (15.6)	< 0.001
Kidney disease	222 (5.7)	74 (5.2)	101 (5.2)	239 (5.6)	0.79
Lung disease	338 (8.7)	133 (9.2)	180 (9.3)	468 (10.9)	0.006
Cancer	31 (0.8)	10 (0.7)	14 (0.7)	40 (0.9)	0.74
Hypoglycemic medication	80 (2.1)	37 (2.6)	56 (2.9)	279 (6.5)	< 0.001
Lipid-lowering medication	88 (2.3)	30 (2.1)	47 (2.5)	399 (9.4)	< 0.001
Antihypertensive medication	—	—	—	2144 (49.9)	—
Depressive symptoms	1497 (38.4)	522 (36.1)	639 (32.8)	1600 (37.2)	< 0.001
BMI, kg/m^2^	22.5 ± 3.2	23.1 ± 3.4	23.6 ± 3.6	24.5 ± 3.8	< 0.001
SBP, mm Hg	109.2 ± 7.3	124.5 ± 2.7	131.2 ± 6.1	149.1 ± 19.4	< 0.001
DBP, mm Hg	65.6 ± 7.1	72.0 ± 5.6	79.7 ± 6.4	84.2 ± 12.0	< 0.001
PP, mm Hg	43.8 ± 6.5	52.8 ± 5.8	51.7 ± 9.9	65.1 ± 16.6	< 0.001

Data are mean ± SD or n (%).

BMI = body-mass index; SBP = systolic blood pressure; DBP = diastolic blood pressure; PP = pulse pressure.

### Association between baseline BP exposure and cognitive decline

**Fig. 2:**
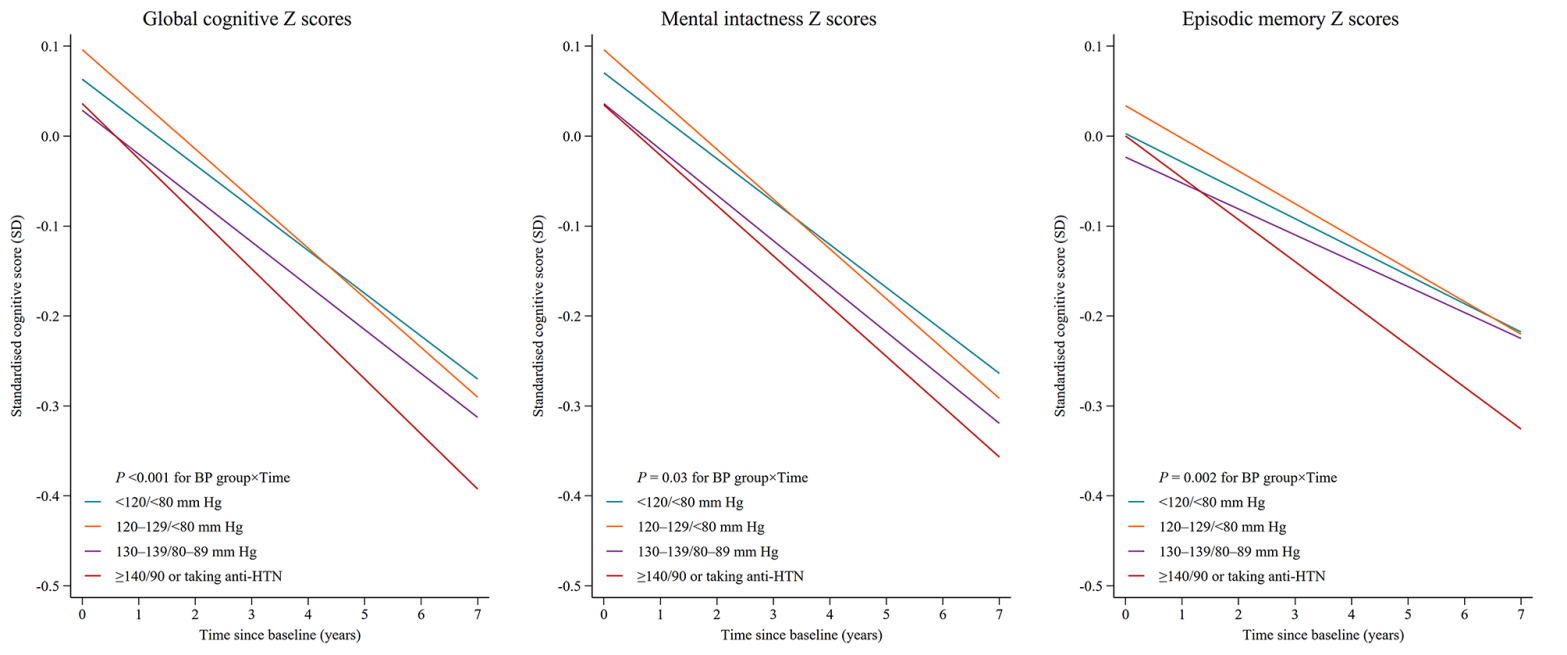
Cognitive trajectories during follow-up according to the blood pressure categories. Mixed linear regression models with random intercepts and slopes were adjusted for age, age^2^, sex, education, marital status, residence, health insurance, smoking status, drinking status, history of cardiovascular diseases, diabetes, dyslipidemia, kidney disease, lung disease, and cancer, depressive symptoms, hypoglycemic medication, lipid-lowering medication, BMI, and BMI^2^. *P* values were calculated for the interaction between blood pressure categories and follow-up time as the timescale. BMI = body-mass index

Table 2; Fig. 2 shows the longitudinal associations between BP categories and rates of change in cognitive function after multivariable adjustment. Compared to individuals with SBP/DBP < 120/80 mm Hg, those with SBP/DBP ≥ 140/90 mm Hg or taking anti-hypertensive medication had significantly faster rates of decline in global cognitive function (-0.014 SD/year; 95% CI: -0.020 to -0.007; *P* < 0.001), mental intactness (-0.008 SD/year; 95% CI: -0.014 to -0.003; *P* = 0.004), and episodic memory (-0.015 SD/year; 95% CI: -0.024 to -0.006; *P* = 0.001).

**Table 2:** Association between blood pressure categories and rate of cognitive decline (SD/Year) over 7 years of follow-up, estimated by linear mixed effects regression

	Population at risk	Model 1*			Model 2†	
		Mean Difference (95% CI) in Rate of Change (SD/Year)	*P* value		Mean Difference (95% CI) in Rate of Change (SD/Year)	*P* value
Global cognitive *Z* scores						
SBP/DBP, mm Hg						
<120/<80	3901	0 (Reference)			0 (Reference)	
120–129/<80	1444	-0.007 (-0.016 to 0.002)	0.11		-0.008 (-0.016 to 0.001)	0.09
130–139/80–89	2026	0.0003 (-0.007 to 0.008)	0.98		-0.001 (-0.009 to 0.007)	0.77
≥140/90 or taking anti-HTN	4300	-0.013 (-0.020 to -0.007)	< 0.001		-0.014 (-0.020 to -0.007)	< 0.001
Test for trend			< 0.001			< 0.001
Mental intactness *Z* scores						
SBP/DBP, mm Hg						
<120/<80	3901	0 (Reference)			0 (Reference)	
120–129/<80	1444	-0.006 (-0.014 to 0.001)	0.11		-0.008 (-0.015 to 0.0001)	0.05
130–139/80–89	2026	-0.002 (-0.009 to 0.005)	0.63		-0.003 (-0.010 to 0.004)	0.40
≥140/90 or taking anti-HTN	4300	-0.008 (-0.013 to -0.002)	0.005		-0.008 (-0.014 to -0.003)	0.004
Test for trend			0.01			0.01
Episodic memory *Z* scores						
SBP/DBP, mm Hg						
<120/<80	3901	0 (Reference)			0 (Reference)	
120–129/<80	1444	-0.005 (-0.018 to 0.007)	0.39		-0.005 (-0.017 to 0.008)	0.45
130–139/80–89	2026	0.004 (-0.007 to 0.015)	0.52		0.003 (-0.008 to 0.014)	0.66
≥140/90 or taking anti-HTN	4300	-0.016 (-0.025 to -0.007)	0.001		-0.015 (-0.024 to -0.006)	0.001
Test for trend			0.002			0.003

Data are adjusted rate of cognitive Z-score decline (SD/year), unless otherwise stated. Anti-HTN = anti-hypertensive medication; BMI = body-mass index;

SBP = systolic blood pressure; DBP = diastolic blood pressure.

*Model 1: adjusted for age, age^2^ and sex.

†Model 2: adjusted for age, age^2^, sex, education, marital status, residence, health insurance, smoking status, alcohol status, history of cardiovascular diseases, diabetes, dyslipidemia, kidney disease, lung disease, and cancer, depressive symptoms, hypoglycemic medication, lipid-lowering medication, BMI, and BMI^2^.

Table 3 shows the association of SBP, DBP, and PP categories with rate of cognitive decline stratified by anti-hypertensive medication status. Individuals with SBP ≥ 140 mm Hg experienced a faster rate of global cognitive decline when compared to those with SBP < 120 mm Hg, irrespective of whether they were taking anti-hypertensive medication (*β*=-0.024 SD/year; 95% CI: -0.032 to -0.016; *P* < 0.001 vs. *β*=-0.017 SD/year; 95% CI: -0.027 to -0.007; *P* < 0.001). Individuals who achieved control of SBP < 140 mm Hg with the use of anti-hypertensive treatment did not demonstrate any association with cognitive decline. Moreover, individuals who achieved control of SBP < 120 mm Hg with the use of anti-hypertensive treatment did not increase the risk of global cognitive decline (*β*=-0.003 SD/year; 95% CI: -0.021 to 0.014; *P* = 0.71). However, participants with elevated DBP were not associated with cognitive decline irrespective of whether they were taking anti-hypertensive medication (Table 3).

**Table 3:** Association between SBP, DBP, PP and rate of cognitive decline (SD/Year) over 7 years of follow-up by anti-htn status, estimated by linear mixed effects regression

		Mean Difference (95% CI) in Rate of Change (SD/Year)*							
	Population at risk	Global cognitive *Z*-scores	*P* value		Mental intactness *Z*-scores	*P* value		Episodic memory *Z*-scores	*P* value
**SBP**									
Not taking anti-HTN									
< 120 (normal SBP)	4018	0 (Reference)			0 (Reference)			0 (Reference)	
120–129	2026	-0.005 (-0.012 to 0.003)	0.24		-0.007 (-0.014 to 0.0001)	0.06		0.0003 (-0.011 to 0.011)	0.96
130–139	1487	-0.003 (-0.012 to 0.006)	0.51		-0.001 (-0.009 to 0.007)	0.78		-0.004 (-0.016 to 0.009)	0.55
≥ 140	1996	-0.024 (-0.032 to -0.016)	< 0.001		-0.013 (-0.020 to -0.006)	< 0.001		-0.033 (-0.045 to -0.022)	< 0.001
Taking anti-HTN									
< 120 (normal SBP)	277	-0.003 (-0.021 to 0.014)	0.71		0.007 (-0.009 to 0.022)	0.41		-0.008 (-0.032 to 0.017)	0.55
120–129	303	0.004 (-0.013 to 0.021)	0.65		-0.001 (-0.016 to 0.013)	0.85		0.017 (-0.006 to 0.041)	0.15
130–139	395	0.005 (-0.011 to 0.020)	0.54		-0.007 (-0.020 to 0.007)	0.34		0.020 (-0.001 to 0.042)	0.07
≥ 140	1169	-0.017 (-0.027 to -0.007)	0.001		-0.008 (-0.017 to 0.0003)	0.06		-0.018 (-0.032 to -0.004)	0.01
Test for trend			0.001			0.04			0.02
**DBP**									
Not taking anti-HTN									
< 80 (normal DBP)	6744	0 (Reference)			0 (Reference)			0 (Reference)	
80–89	1925	0.003 (-0.005 to 0.010)	0.46		-0.002 (-0.009 to 0.004)	0.52		0.008 (-0.002 to 0.019)	0.13
≥ 90	858	-0.005 (-0.016 to 0.005)	0.33		-0.007 (-0.016 to 0.002)	0.15		-0.006 (-0.020 to 0.009)	0.47
Taking anti-HTN									
80 (normal DBP)	976	-0.004 (-0.014 to 0.006)	0.43		0.001 (-0.008 to 0.010)	0.87		-0.002 (-0.016 to 0.012)	0.78
80–89	638	-0.003 (-0.015 to 0.010)	0.67		-0.007 (-0.018 to 0.004)	0.19		0.006 (-0.011 to 0.023)	0.48
≥ 90	530	0.005 (-0.009 to 0.018)	0.51		-0.0002 (-0.012 to 0.012)	0.97		0.014 (-0.005 to 0.033)	0.14
Test for trend			0.73			0.37			0.33
**PP**									
Not taking anti-HTN									
< 50 (normal PP)	4700	0 (Reference)			0 (Reference)			0 (Reference)	
50–59	2627	-0.013 (-0.020 to -0.006)	< 0.001		-0.007 (-0.013 to -0.001)	0.02		-0.017 (-0.026 to -0.007)	0.001
60–69	1244	-0.023 (-0.032 to -0.014)	< 0.001		-0.008 (-0.016 to 0.0002)	0.06		-0.033 (-0.046 to -0.020)	0.001
≥ 70	956	-0.038 (-0.048 to -0.027)	0.001		-0.011 (-0.020 to -0.002)	0.02		-0.065 (-0.080 to -0.050)	0.001
Taking anti-HTN									
< 50 (normal PP)	482	0.005 (-0.009 to 0.018)	0.48		0.002 (-0.010 to 0.014)	0.79		0.011 (-0.008 to 0.030)	0.24
50–59	566	-0.004 (-0.017 to 0.009)	0.52		-0.004 (-0.015 to 0.007)	0.50		0.003 (-0.015 to 0.021)	0.74
60–69	443	-0.012 (-0.027 to 0.002)	0.10		-0.009 (-0.022 to 0.004)	0.16		-0.005 (-0.025 to 0.016)	0.65
≥ 70	653	-0.033 (-0.045 to -0.020)	< 0.001		-0.008 (-0.019 to 0.003)	0.16		-0.051 (-0.069 to -0.033)	< 0.001
Test for trend			0.001			0.07			0.001

Data are adjusted rate of cognitive Z-score decline (SD/year), unless otherwise stated. Anti-HTN = antihypertensive medication; BMI = body-mass index; SBP=systolic blood pressure; DBP = diastolic blood pressure; PP = pulse pressure.

*Model adjusted for age, age^2^, sex, education, marital status, residence, health insurance, smoking status, alcohol status, history of cardiovascular diseases, diabetes, dyslipidemia, kidney disease, lung disease, and cancer, depressive symptoms, hypoglycemic medication, lipid-lowering medication, BMI, and BMI^2^.

Of 2,144 participants taking anti-hypertensive medication, 1,491 (69.5%) had PP controlled to less than 70 mm Hg (Table 3). Among those not taking anti-hypertensive treatment, individuals with PP ≥ 50 mm Hg experienced a greater rate of global cognitive decline compared to those with PP < 50 mm Hg (Table 3). Participants taking anti-hypertensive and with PP ≥ 70 mm Hg had significantly higher rate of cognitive decline than did those without taking anti-hypertensive and with PP < 50 mm Hg (*β*=-0.033 SD/year; 95% CI: -0.045 to -0.020; *P* < 0.001). Moreover, individuals who achieved control of PP < 70 mm Hg with the use of anti-hypertensive treatment did not demonstrate any association with cognitive decline.

### Does-response relationship of baseline BP and cognitive decline

**Fig. 3:**
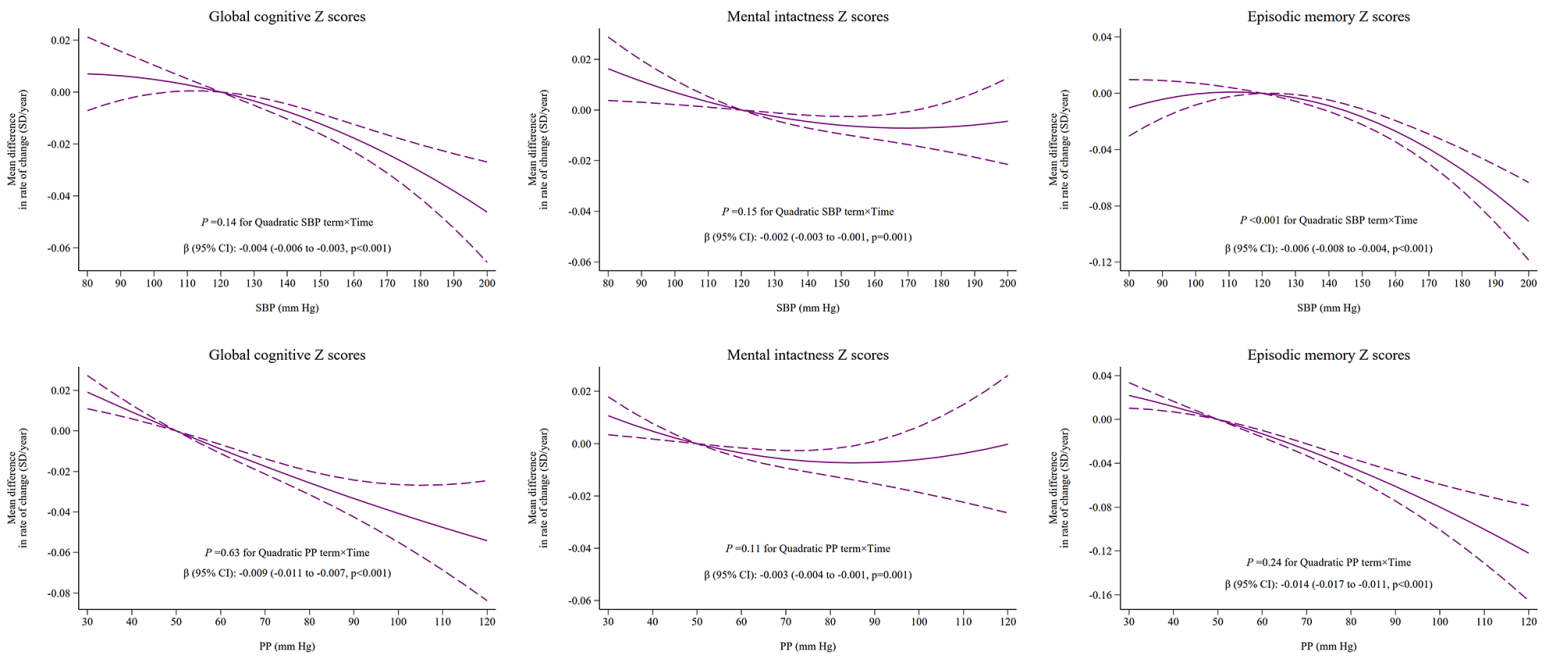
Dose-response curves of SBP, PP and rate of cognitive decline (SD/year) mixed linear regression models with random intercepts and slopes were adjusted for age, age^2^, sex, education, marital status, residence, health insurance, smoking status, drinking status, history of cardiovascular diseases, diabetes, dyslipidemia, kidney disease, lung disease, and cancer, depressive symptoms, hypoglycemic medication, lipid-lowering medication, and anti-hypertensive medication, BMI, and BMI^2^. BMI = body-mass index; SBP = systolic blood pressure; PP = pulse pressure

The rate of cognitive decline was significantly associated with elevated SBP and with elevated PP (Fig. 3). Overall, an increase in every 10 mm Hg of SBP was associated with a greater rate of decline in global cognitive function of -0.004 SD/year (95% CI: -0.006 to -0.003; *P* < 0.001), mental intactness of -0.002 SD/year (95% CI: -0.003 to -0.001; *P* = 0.001), and episodic memory of -0.006 SD/year (95% CI: -0.008 to -0.004; *P* < 0.001), respectively. An increase in every 10 mm Hg of PP was associated with an accelerated decline rate in global cognitive function of -0.009 SD/year (95% CI: -0.011 to -0.007; *P* < 0.001), mental intactness of -0.003 SD/year (95% CI: -0.004 to -0.001; *P* = 0.001), and episodic memory of -0.014 SD/year (95% CI: -0.017 to -0.011; *P* < 0.001), respectively. However, elevated DBP was not significantly associated with cognitive decline (Fig. S1 in Supplement).

### Association between cumulative BP exposure and cognitive decline

The mean cumulative exposure to SBP, DBP, and PP was 258.3 ± 36.6, 150.3 ± 21.0, and 107.8 ± 25.5 mm Hg×years, respectively. As shown in Table 4 and Fig. S2 in the Supplement, higher cumulative SBP and PP were associated with accelerated cognitive decline, while higher cumulative DBP was not associated with accelerated cognitive decline. Compared with the lowest tertile, participants in the highest tertile of cumulative SBP and PP had accelerated global cognitive decline of -0.029 SD/year (95% CI: -0.041 to -0.017; *P* < 0.001) and − 0.046 SD/year (95% CI: -0.058 to -0.033; *P* < 0.001), respectively. Each SD increment was associated with an accelerated global cognitive decline of -0.012 SD/year (95% CI: -0.017 to -0.007; *P* = 0.001) for cumulative SBP and -0.017 SD/year (95% CI: -0.022 to -0.012; *P* < 0.001) for cumulative PP. The associations of cumulative BP with rate of decline in global cognitive function and mental intactness did not vary by anti-hypertensive medication status (Table 4).

**Table 4:** Association between cumulative blood pressure exposure and cognitive decline rate (SD/Year) over 5 years of follow-up, estimated by linear mixed effects regression

		Mean Difference (95% CI) in Rate of Change (SD/Year)*							
	Population at risk	Global cognitive *Z*-scores	*P* value		Mental intactness *Z*-scores	*P* value		Episodic memory *Z*-scores	*P* value
Cumulative SBP, mm Hg × years									
Tertile 1	2661	0 (Reference)			0 (Reference)			0 (Reference)	
Tertile 2	2642	-0.017 (-0.029 to -0.005)	0.006		-0.013 (-0.023 to -0.003)	0.01		-0.012 (-0.029 to 0.005)	0.16
Tertile 3	2622	-0.029 (-0.041 to -0.017)	< 0.001		-0.015 (-0.026 to -0.004)	0.005		-0.036 (-0.053 to -0.018)	< 0.001
Test for linear trend			< 0.001			0.005			< 0.001
Per SD increment									
Overall	7925	-0.012 (-0.017 to -0.007)	0.001		-0.005 (-0.009 to -0.001)	0.02		-0.017 (-0.024 to -0.010)	0.001
Not taking anti-HTN	5956	-0.012 (-0.019 to -0.006)	0.001		-0.007 (-0.013 to -0.001)	0.01		-0.014 (-0.024 to -0.005)	0.004
Taking anti-HTN	1969	-0.015 (-0.025 to -0.005)	0.003		-0.002 (-0.011 to 0.007)	0.69		-0.031 (-0.046 to -0.017)	0.001
Cumulative DBP, mm Hg × years									
Tertile 1	2657	0 (Reference)			0 (Reference)				
Tertile 2	2628	-0.005 (-0.017 to 0.007)	0.44		-0.003 (-0.013 to 0.008)	0.61		-0.001 (-0.019 to 0.016)	0.89
Tertile 3	2640	-0.002 (-0.014 to 0.010)	0.73		-0.004 (-0.015 to 0.006)	0.42		0.003 (-0.015 to 0.021)	0.74
Test for linear trend			0.72			0.42			0.74
Per SD increment									
Overall	7925	-0.001 (-0.006 to 0.003)	0.42		-0.001 (-0.006 to 0.003)	0.52		0.002 (-0.005 to 0.009)	0.63
Not taking anti-HTN	5956	0.001 (-0.006 to 0.007)	0.82		-0.001 (-0.007 to 0.004)	0.64		0.004 (-0.006 to 0.013)	0.45
Taking anti-HTN	1969	0.002 (-0.008 to 0.011)	0.73		-0.001 (-0.009 to 0.008)	0.86		0.002 (-0.012 to 0.016)	0.75
Cumulative PP, mm Hg × years									
Tertile 1	2689	0 (Reference)			0 (Reference)			0 (Reference)	
Tertile 2	2606	-0.022 (-0.034 to -0.010)	< 0.001		-0.009 (-0.019 to 0.002)	0.11		-0.030 (-0.047 to -0.013)	0.001
Tertile 3	2630	-0.046 (-0.058 to -0.033)	< 0.001		-0.019 (-0.029 to -0.008)	< 0.001		-0.064 (-0.082 to -0.047)	< 0.001
Test for linear trend			< 0.001			< 0.001			< 0.001
Per SD increment									
Overall	7925	-0.017 (-0.022 to -0.012)	< 0.001		-0.006 (-0.011 to -0.002)	0.006		-0.026 (-0.034 to -0.019)	< 0.001
Not taking anti-HTN	5956	-0.019 (-0.025 to -0.012)	< 0.001		-0.009 (-0.015 to -0.004)	0.002		-0.024 (-0.034 to -0.015)	< 0.001
Taking anti-HTN	1969	-0.018 (-0.027 to -0.010)	< 0.001		-0.002 (-0.010 to 0.006)	0.71		-0.036 (-0.049 to -0.023)	0.001

Data are adjusted rate of cognitive Z-score decline (SD/year), unless otherwise stated. BMI = body-mass index; SBP = systolic blood pressure; DBP = diastolic blood pressure; PP = pulse pressure.

*Model adjusted for age, age^2^, sex, education, marital status, residence, health insurance, smoking status, alcohol status, history of cardiovascular diseases, diabetes, dyslipidemia, kidney disease, lung disease, and cancer, depressive symptoms, hypoglycemic medication, lipid-lowering medication, anti-hypertensive medication, BMI, and BMI^2^.

### Sensitivity analyses

Baseline age significantly modified the relationships of baseline BP categories with global cognitive decline (*P* < 0.001 for age×BP×time, Fig. S3 in Supplement). The association between baseline BP categories, cumulative BP and global cognitive decline was robust and stable in males and females (Fig. S4 and Table S1 in Supplement). Similar findings were observed when excluding individuals with pre-existing cardiovascular diseases, diabetes, or both at baseline (Fig. S5 and Table S2 in Supplement). The dose-response relationship between baseline SBP, PP, and global cognitive decline did not vary according to anti-hypertensive treatment status (Fig. S6 in Supplement). Similar findings were observed when using more categories of SBP, DBP, and PP (Fig. S7-S9 in Supplement).

## Discussion

In this population-based cohort of Chinese middle-aged and older adults, we had several findings. First, during a maximum follow-up of 7 years, those with SBP ≥ 140 mm Hg irrespective of whether they were taking anti-hypertensive medications were at significantly higher risk of cognitive decline than those with normotension. Second, participants with hypertension treated to an attained SBP < 120 mm Hg did not have a significantly increased risk of cognitive decline compared with those with normotension. Third, participants who were not on anti-hypertensive treatment and who had a PP ≥ 50 mm Hg, or those on anti-hypertensive treatment and had a PP ≥ 70 mm Hg were at significantly higher risk of cognitive decline than were those with PP < 50 mm Hg. Finally, cumulative exposure to SBP and PP was associated with subsequent cognitive decline.

We observed a significant association between SBP/DBP ≥ 140/90 mm Hg or taking anti-hypertensive medication, and SBP ≥ 140 mm Hg (regardless of anti-hypertensive medication status) with risk of cognitive decline. These findings are consistent with several prior reports [5–9]. For example, findings from the Atherosclerosis Risk in Communities (ARIC) study in a cohort of 13,476 participants, showed that participants with midlife hypertension (SBP ≥ 140 or DBP ≥ 90 mm Hg or antihypertensive use) or those with elevated SBP (≥ 140 mm Hg) had a greater likelihood of cognitive decline over 20 years of follow-up [5]. Therefore, treating hypertension by implementing a cutoff of SBP of 140 mm Hg may prove to be an effective strategy in mitigating the risk of cognitive decline in Chinese middle-aged and older adults.

Our data suggests that that Chinese middle-aged and older adults with hypertension who were able to achieve a SBP of less than 120 mm Hg had no significantly increased risk of cognitive decline compared to individuals with normotension. In contrast, a secondary analysis from the SPRINT suggested that intensive control of SBP to a target of 120 mm Hg had neither a beneficial or detrimental effect on specific domains of cognitive decline [28]. On the other hand, intensive BP control was associated with a lower development of mild cognitive impairment but not for probable dementia in SPRINT [4], though this finding was not replicated in a systematic review and meta-analysis that included 4 additional randomized controlled trials with intensive BP targets [29], though BP lowering in general is associated with lower risk of incident dementia [30]. Further long-term observational and randomized controlled trial evidence is needed.

Several studies examined the association between high PP and risk of cognitive decline [7, 31–33]. We first observed a significant does-response association of elevated PP with risk of cognitive decline irrespective of anti-hypertensive medication status. This is consistent with findings from the REGARDS (Reasons for Geographic and Racial Differences in Stroke) cohort, which included 22,164 black and white individuals aged 45 years and older, with a median follow-up period of 8.1 years [33]. In addition, we observed that PP ≥ 50 mm Hg and not taking anti-hypertensive medication as well as PP ≥ 70 mm Hg and taking anti-hypertensive medication were both associated with an increased rate of cognitive decline. This significant result might partly be attributable to the effect of anti-hypertensive treatment on the threshold of PP with risk of cognitive decline. Since antihypertensive treatment could have neuroprotective properties, the threshold of PP associated with cognitive decline among individuals undergoing antihypertensive medication might be higher compared to those who are not. We also observed that elevated PP had a larger effect on the rate of global cognitive decline than elevated SBP or DBP. There are several plausible reasons for this finding. First, SBP and DBP increase with age up to approximately 60 years, while SBP continues to increase while DBP starts to decrease, leading to a gradual increase in PP over time [34]. Second, PP serves as a measure of arterial stillness and elevated PP has been linked to cerebral microvascular diseases, which may in turn leads to cognitive decline [35].

The association between DBP and cognition is not firmly established, with mixed finding [36–38]. For example, The REGARDS study showed that a higher DBP was cross-sectionally and independently associated with impaired cognitive status [36], whereas another cross-sectional study revealed that a lower DBP was inversely related to cognitive impairment [37]. Furthermore, a longitudinal cohort of 7874 Chinese individuals aged 60 years or older showed that a lower DBP was associated with greater subsequent cognitive decline over 2-year follow-up [38]. In the present study, we did not observe a significant association between baseline or cumulative DBP and cognitive decline in any of the cognitive domains, irrespective of anti-hypertensive medication status. Furthermore, findings from the SPRINT MIND (Systolic Blood Pressure Intervention Trial Memory and Cognition in Decreased Hypertension) showed that intensive SBP lowering did not result in harm to cognitive outcomes, regardless of baseline DBP levels [39]. More research is needed to examine the association between DBP and cognitive decline in elderly people.

In addition to baseline BP levels and cognitive decline, we also observed that elevated cumulative SBP and PP levels were associated with cognitive decline. However, studies of the association of cumulative BP with risk of cognitive decline in older adults are limited [19]. Furthermore, to date, there is a scarcity of research on the association of cumulative BP cognitive decline in Chinese middle-aged and older adults, despite the fact that socioeconomic and contextual differences have different impacts on cognitive decline [40]. Nevertheless, our findings were generally consistent with results from studies conducted in the UK and US [19].

Our study has important public health implications for hypertension screening and control efforts in China and possibly elsewhere. Namely, our findings re-affirm the need for better BP control particularly among individuals with known stage II hypertension, as defined by the most recent ACC/AHA guidelines, to delay/avoid cognitive decline. The adoption of the 2017 ACC/AHA hypertension guidelines in China would classify 266.9 million, or 55% of individuals aged 45–75 years, as having hypertension. Additionally, it is estimated that 129.8 million patients with hypertension are currently untreated, based on current treatment patterns and the 2017 ACC/AHA hypertension guidelines [41]. Therefore, it is crucial to achieve higher levels of awareness and control of BP in order to reduce the burden of cognitive decline and dementia in China in the future.

### Study strengths and limitations

Our study has several strengths which include its longitudinal design, assessment of multiple cognitive domains, large sample size and nationally representative data. Our study also has several limitations. First, our study only included Chinese adults, so generalization of our findings to other racial/ethnic groups may not be possible. Second, although we adjusted for a range of covariates, we could not rule out the possibility of residual confounding by some uncontrolled factors, such as dietary factors and apolipoprotein E4 (*APOE4*). Third, our study had a relatively short follow-up period for assessing changes in cognition related to baseline BP levels (a maximum of 7 years) and cumulative BP exposure (a maximum of 5 years). Future longitudinal studies are necessary to validate our findings in cohorts with extended follow-up periods. Finally, due to the nature of observational studies, our study cannot determine the causal relationship of BP and cognitive decline, though the robustness of our findings to multiple sensitivity analyses and concordant findings from randomized trials and mechanistic studies suggest that confounding is unlikely to entirely explain these associations.

## Conclusions

In conclusion, hypertension, elevated SBP or elevated PP, were associated with a greater rate of cognitive decline compared to those with normotension. Older adults taking anti-hypertensive treatment with SBP lower than 120 mm Hg or with PP less than 50 mm Hg did not experience an increased rate of cognitive decline when compared to normotensive individuals. We also found that cumulative exposure to SBP and PP was associated with subsequent cognitive decline in middle-aged and older Chinese adults. These results add new evidence that for middle-aged and older individuals, effort decreasing SBP and PP levels might have a pivotal role in preserving cognitive decline in later life.

### Electronic supplementary material

Below is the link to the electronic supplementary material.

Supplementary Material 1

## Data Availability

The CHARLS data set is freely available to all bona fide researchers. Researchers can gain access to the data (http://charls.pku.edu.cn/en).
