# Supplementary material for: Association between untreated and treated blood pressure levels and cognitive decline in community-dwelling middle-aged and older adults in China: a longitudinal study
Source: Alzheimers Res Ther. 2024 May 10;16:104. doi: 10.1186/s13195-024-01467-y (PMC11083800; doi:10.1186/s13195-024-01467-y)
Supplement: Supplementary file 1 — Supplementary Material 1 [file 13195_2024_1467_MOESM1_ESM.docx]

Supplement for

“**Association between blood pressure levels and cognitive decline in community-dwelling middle-aged and older adults in China: a longitudinal cohort study**”

1. Figure S1. Does-Response Relationship of Baseline Diastolic Blood Pressure (DBP) and Cognitive Decline Over 7 Years of Follow-Up
2. Figure S2. Association Between Cumulative Blood Pressure Exposure and Trajectories Cognitive Score Over 5 Years of Follow-Up
3. Figure S3. Association Between Blood Pressure Categories and Rate of Global Cognitive Decline (SD/Year) Over 7 Years of Follow-Up with SBP/DBP <120/<80 mm Hg as Reference, by Age
4. Figure S4. Association Between Blood Pressure Categories and Rate of Global Cognitive Decline (SD/Year) Over 7 Years of Follow-Up with SBP/DBP <120/<80 mm Hg as Reference, by Sex
5. Figure S5. Association Between Blood Pressure Categories and Rate of Global Cognitive Decline (SD/Year) Over 7 Years of Follow-Up with SBP/DBP <120/<80 mm Hg as Reference, Excluding Those with Cardiovascular Diseases, Diabetes, or Both at Baseline
6. Figure S6. Association Between SBP, PP and Rate of Global Cognitive Decline (SD/Year) Over 7 Years of Follow-Up, By Anti-HTN Status
7. Figure S7. Association Between SBP and Rate of Global Cognitive Decline (SD/Year) Over 7 Years of Follow-Up, with Additional Adjustment of Anti-Hypertensive Medication Use, Using <120 mmHg as the Reference Group
8. Figure S8. Association Between DBP and Rate of Global Cognitive Decline (SD/Year) Over 7 Years of Follow-Up, with Additional Adjustment of Anti-Hypertensive Medication Use, Using <60 mmHg as the Reference Group
9. Figure S9. Association Between PP and Rate of Global Cognitive Decline (SD/Year) Over 7 Years of Follow-Up, with Additional Adjustment of Anti-Hypertensive Medication Use, Using <40 mmHg as the Reference Group
10. Table S1. Association Between Cumulative Blood Pressure Exposure and Global Cognitive Decline Rate (SD/Year) Over 5 Years of Follow-Up, Estimated by Linear Mixed Effects Regression, by Subgroup
11. Table S2. Association Between Cumulative Blood Pressure Exposure and Global Cognitive Decline Rate (SD/Year) Over 5 Years of Follow-Up, Estimated by Linear Mixed Effects Regression, Excluding Those with Cardiovascular Diseases, Diabetes, or Both at Baseline


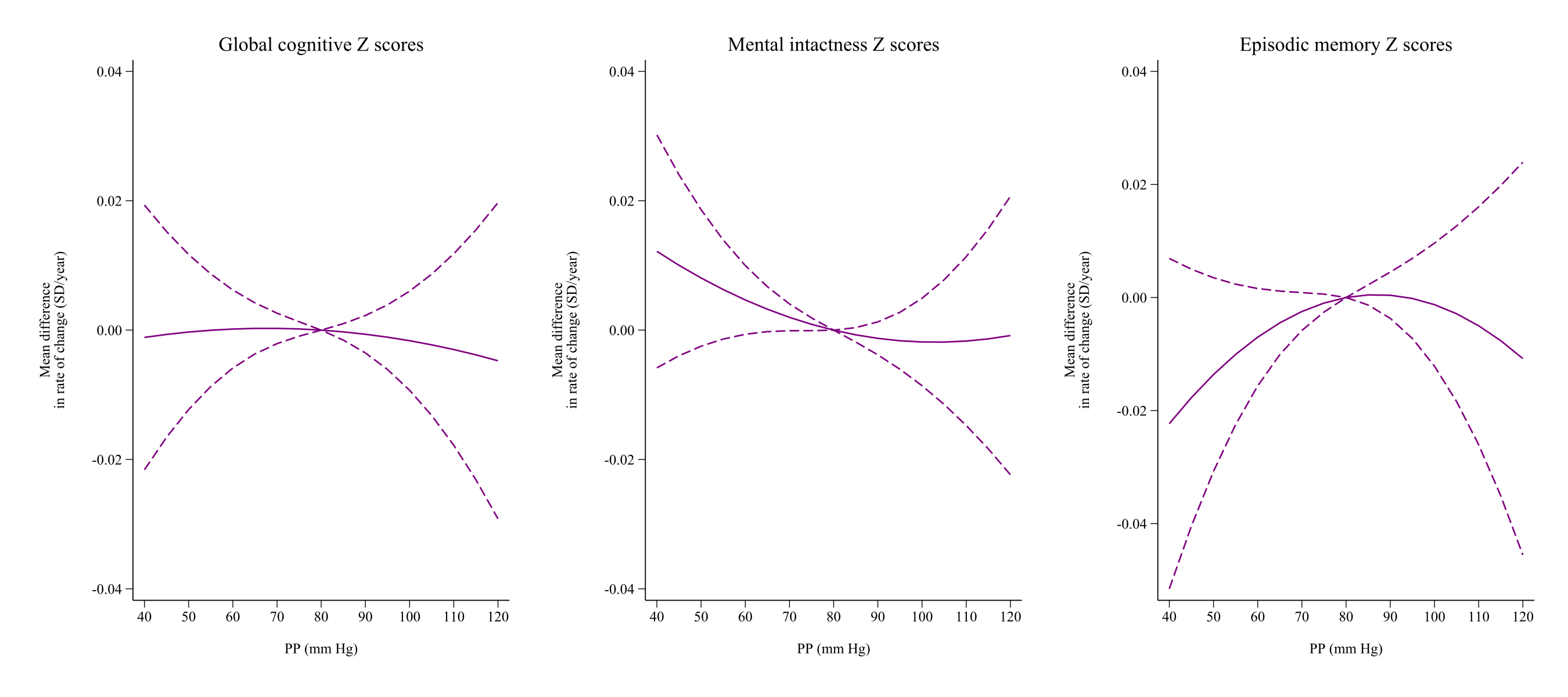


**Figure S1. Does-Response Relationship of Baseline Diastolic Blood Pressure (DBP) and Cognitive Decline Over 7 Years of Follow-Up**

**(A) Global cognitive Z scores**


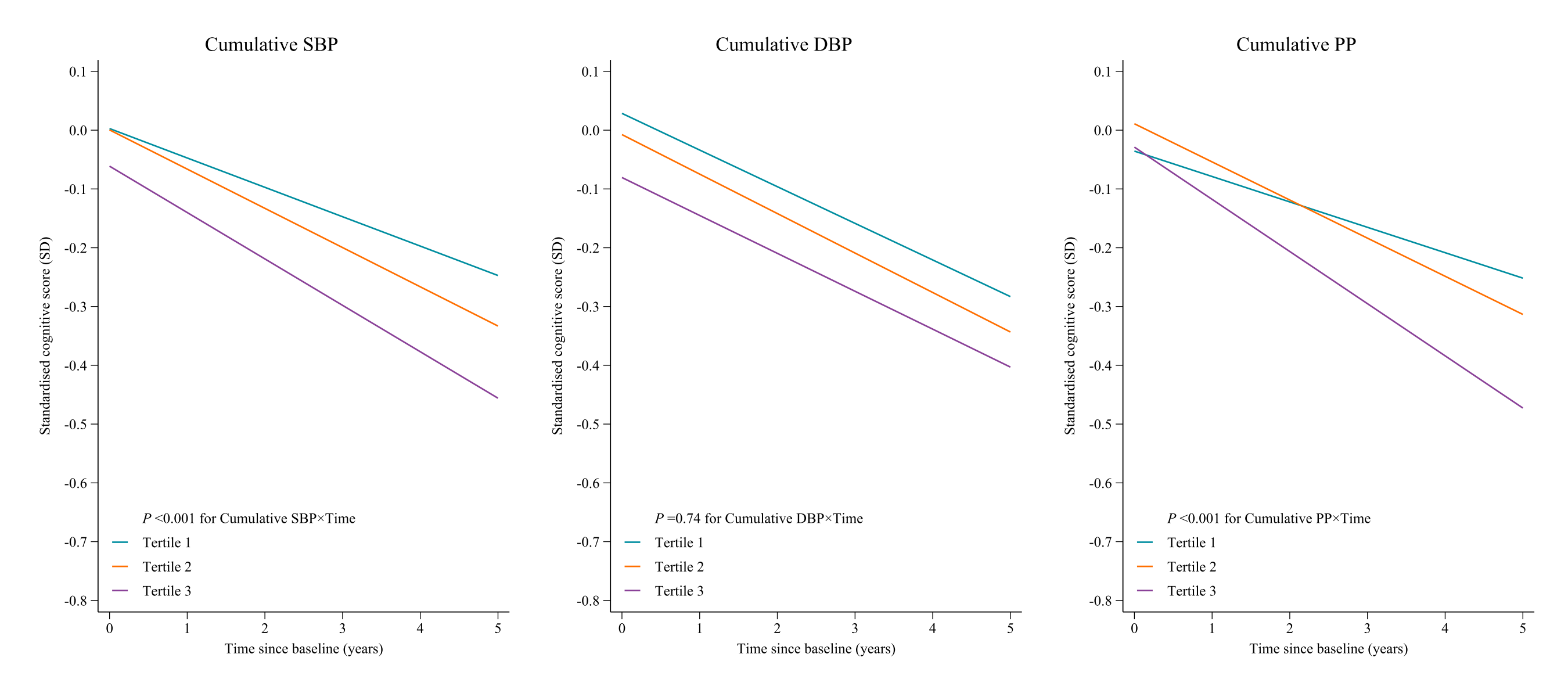


1. **Mental intactness Z scores**


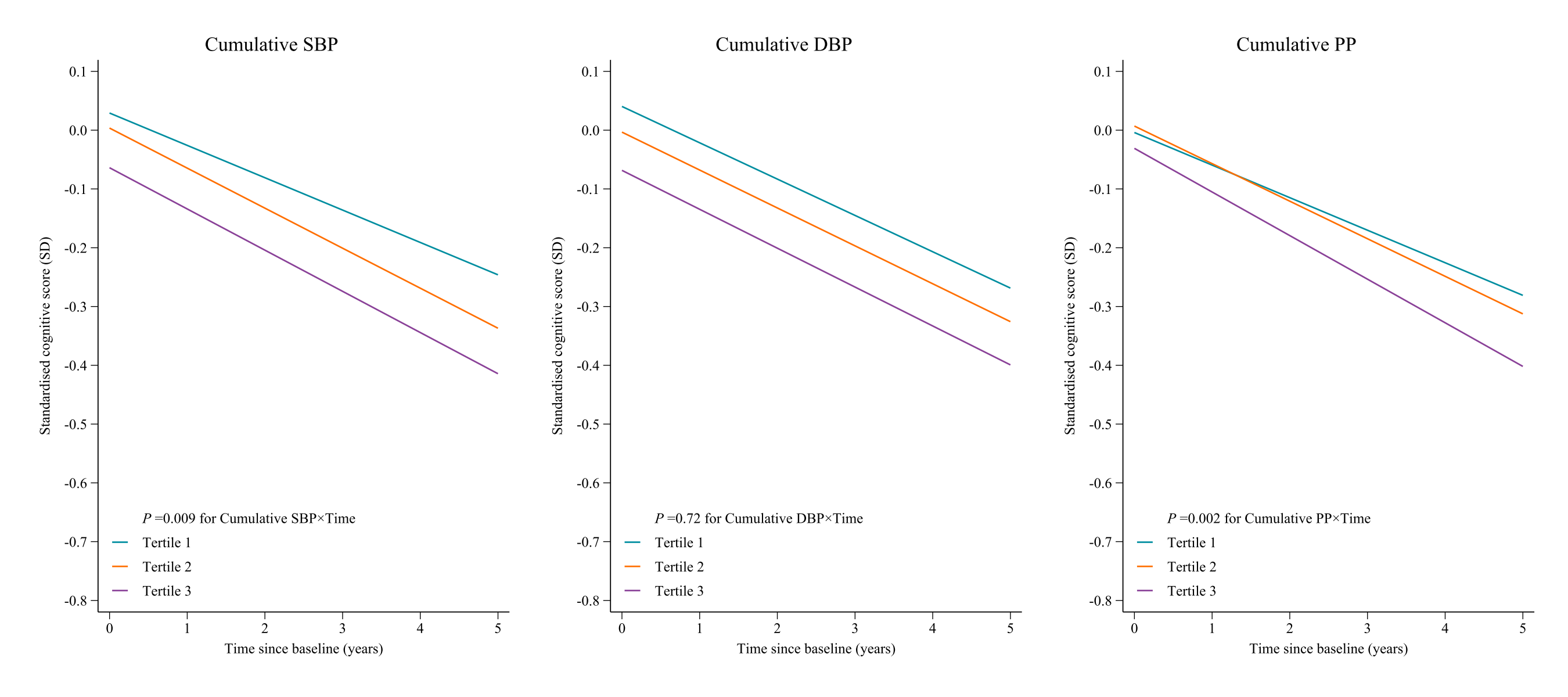


**(C) Episodic memory Z scores**


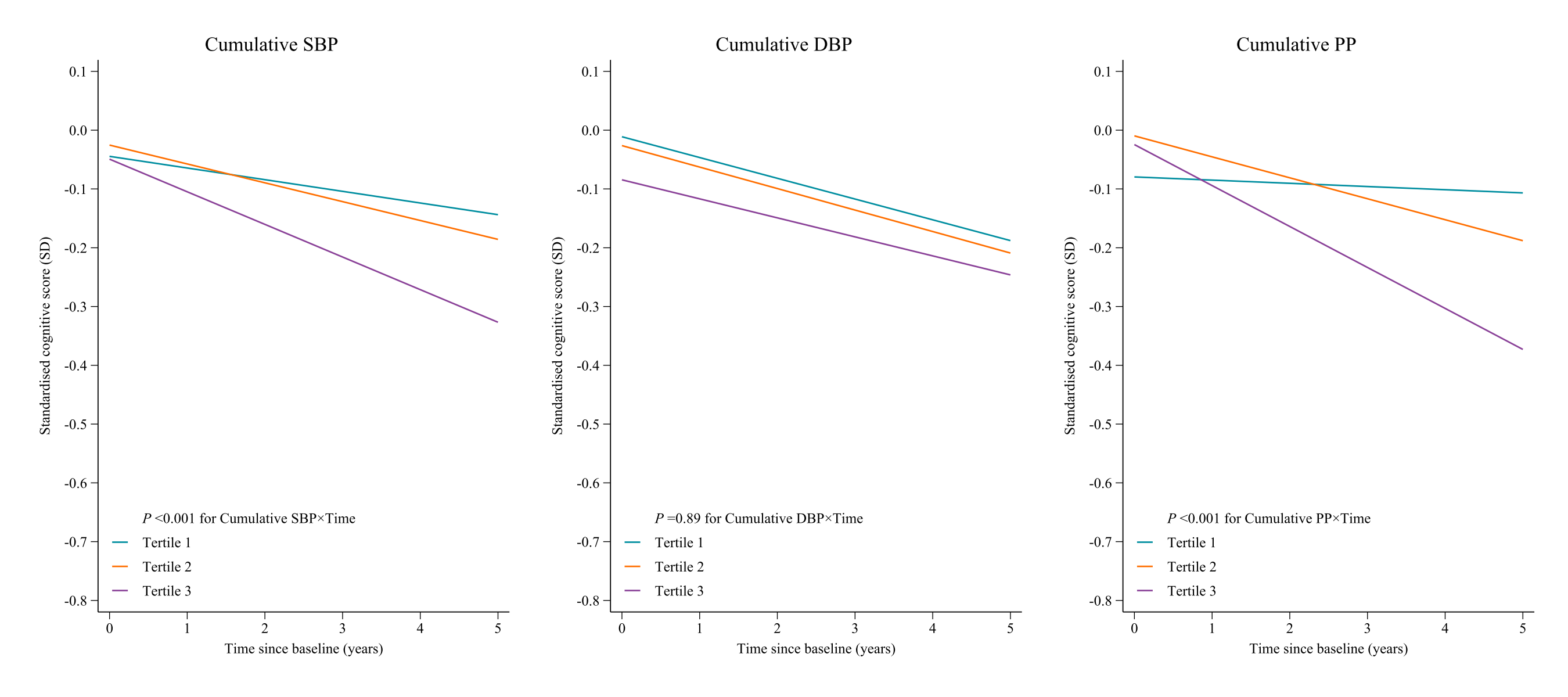


**Figure S2. Association Between Cumulative Blood Pressure Exposure and Trajectories Cognitive Score Over 5 Years of Follow-Up**


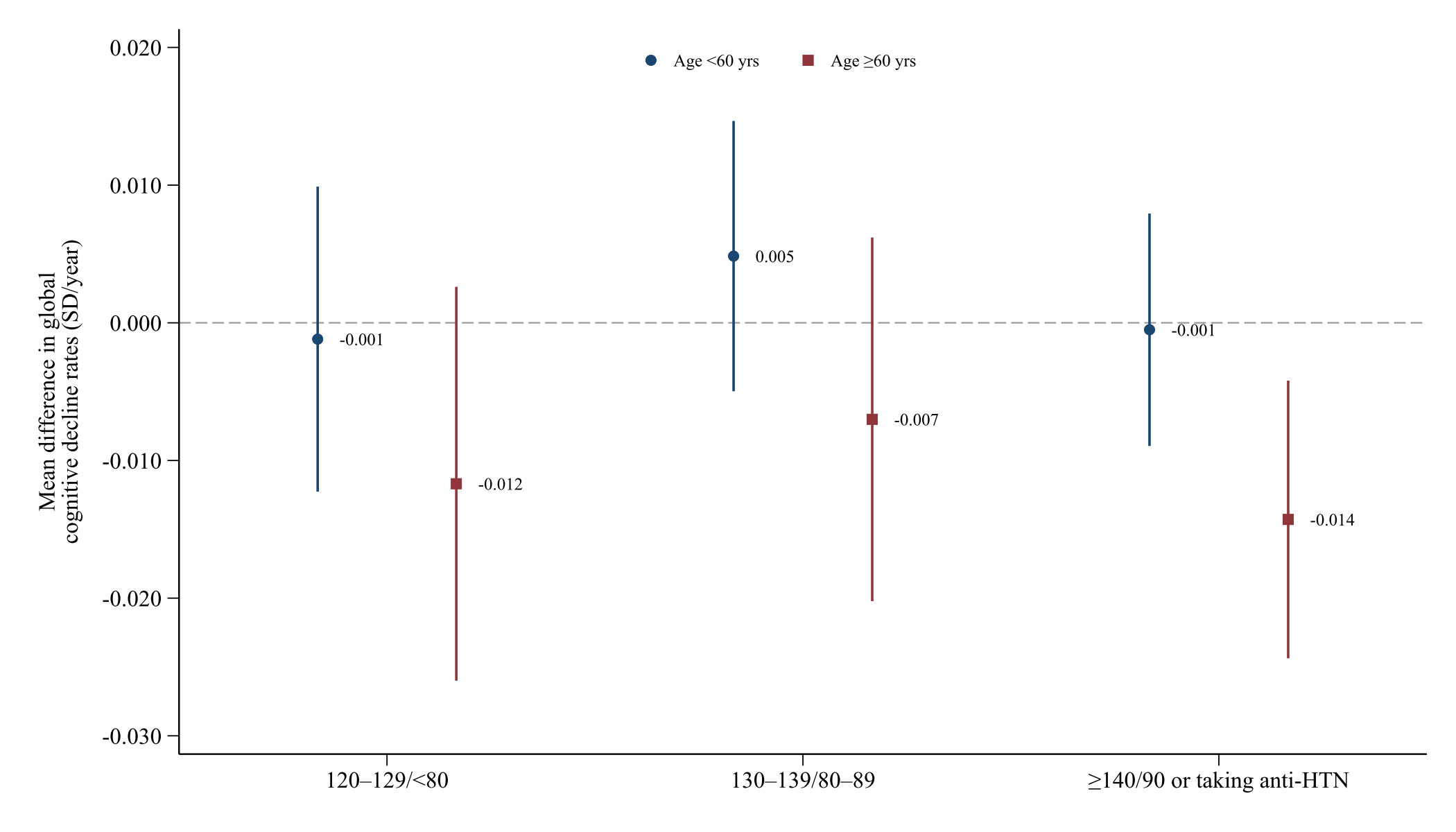


**Figure S3. Association Between Blood Pressure Categories and Rate of Global Cognitive Decline (SD/Year) Over 7 Years of Follow-Up with SBP/DBP <120/<80 mm Hg as Reference, by Age**

The *P* value for the interaction of BP categories×time×age group <0.001 by likelihood-ratio test.


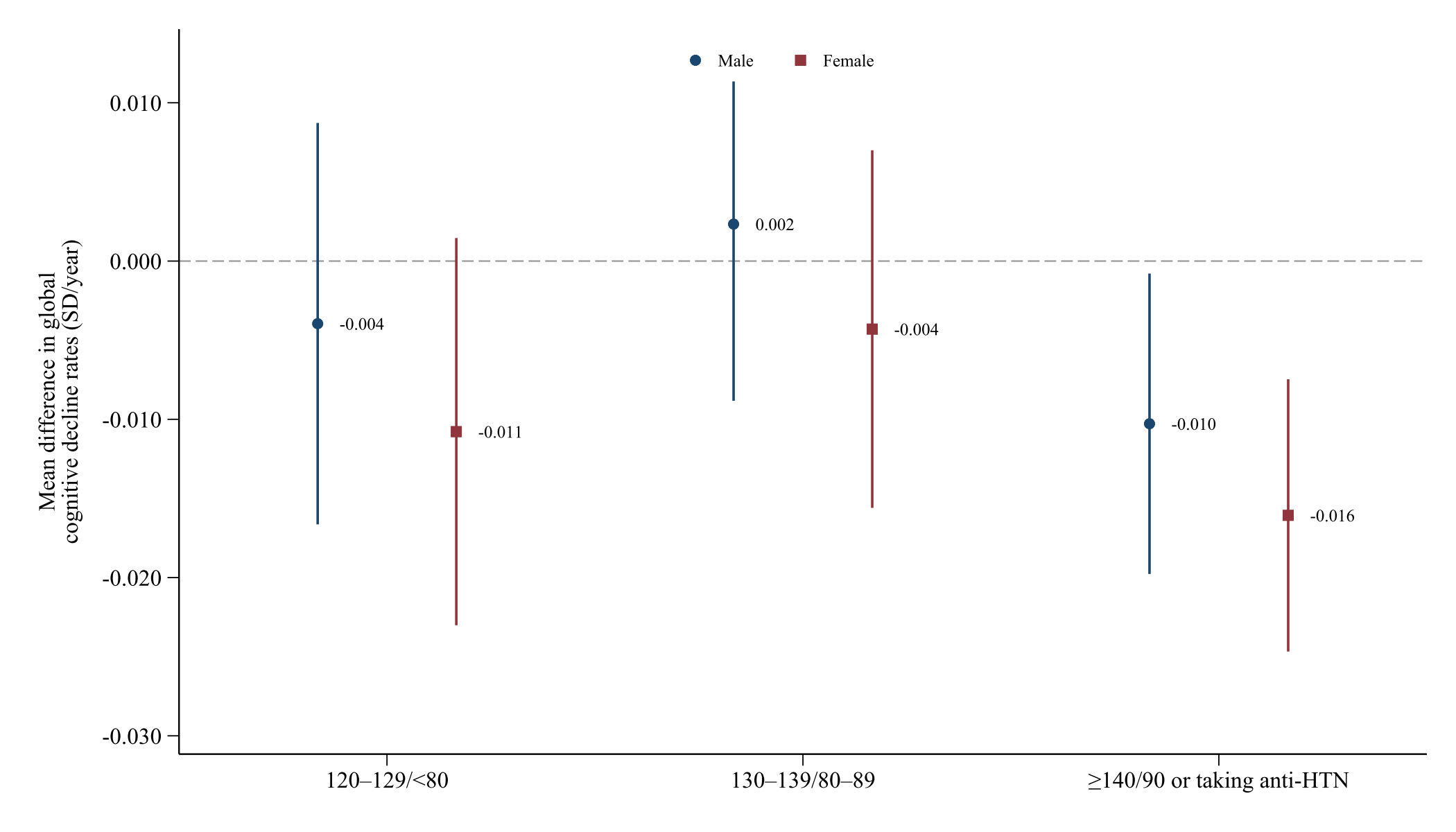


**Figure S4. Association Between Blood Pressure Categories and Rate of Global Cognitive Decline (SD/Year) Over 7 Years of Follow-Up with SBP/DBP <120/<80 mm Hg as Reference, by Sex**

The *P* value for the interaction of BP categories×time×sex = 0.37 by likelihood-ratio test.


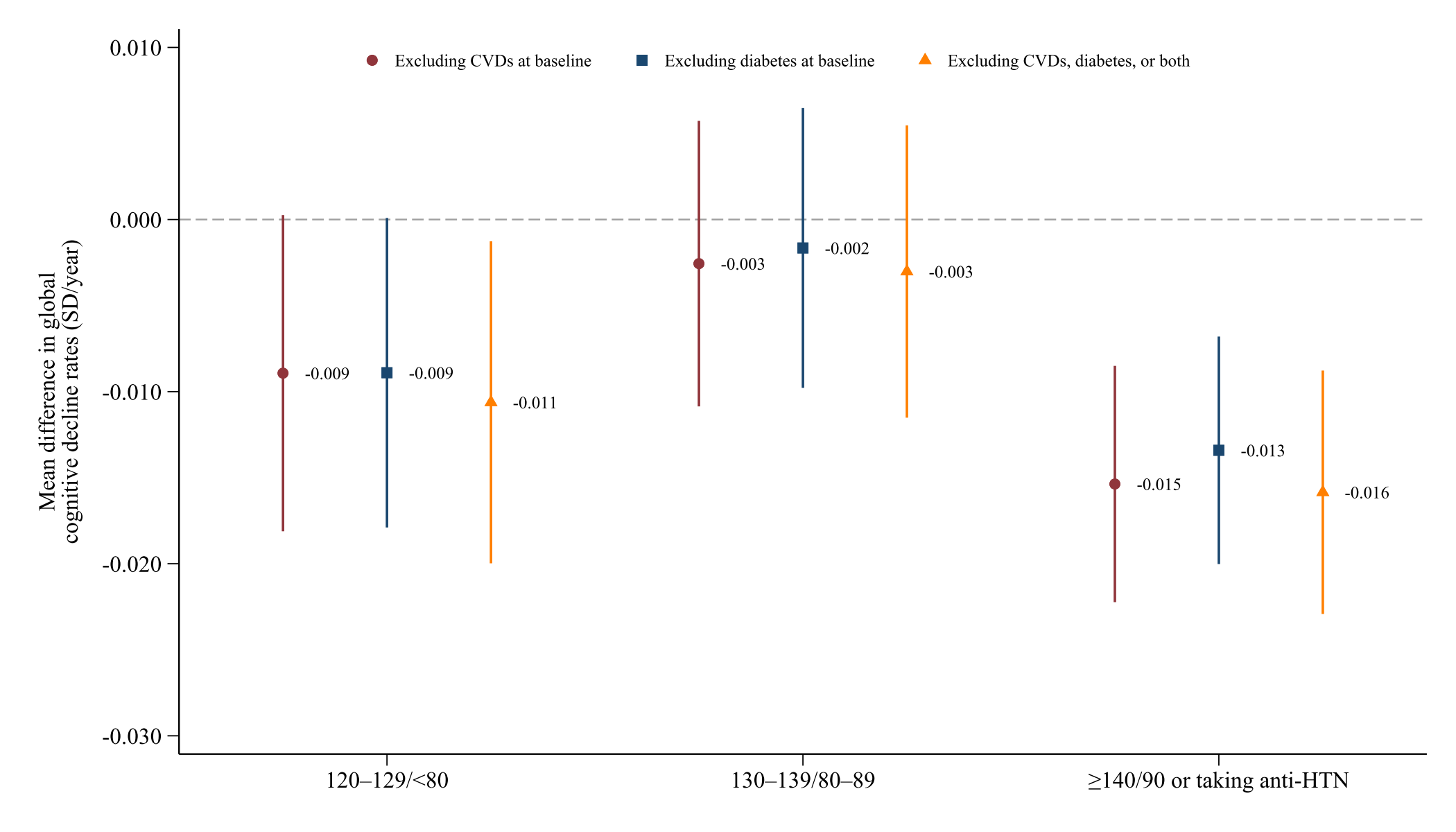


**Figure S5. Association Between Blood Pressure Categories and Rate of Global Cognitive Decline (SD/Year) Over 7 Years of Follow-Up with SBP/DBP <120/<80 mm Hg as Reference, Excluding Those with Cardiovascular Diseases, Diabetes, or Both at Baseline**

1. **SBP and global cognitive Z scores by anti-HTN status**


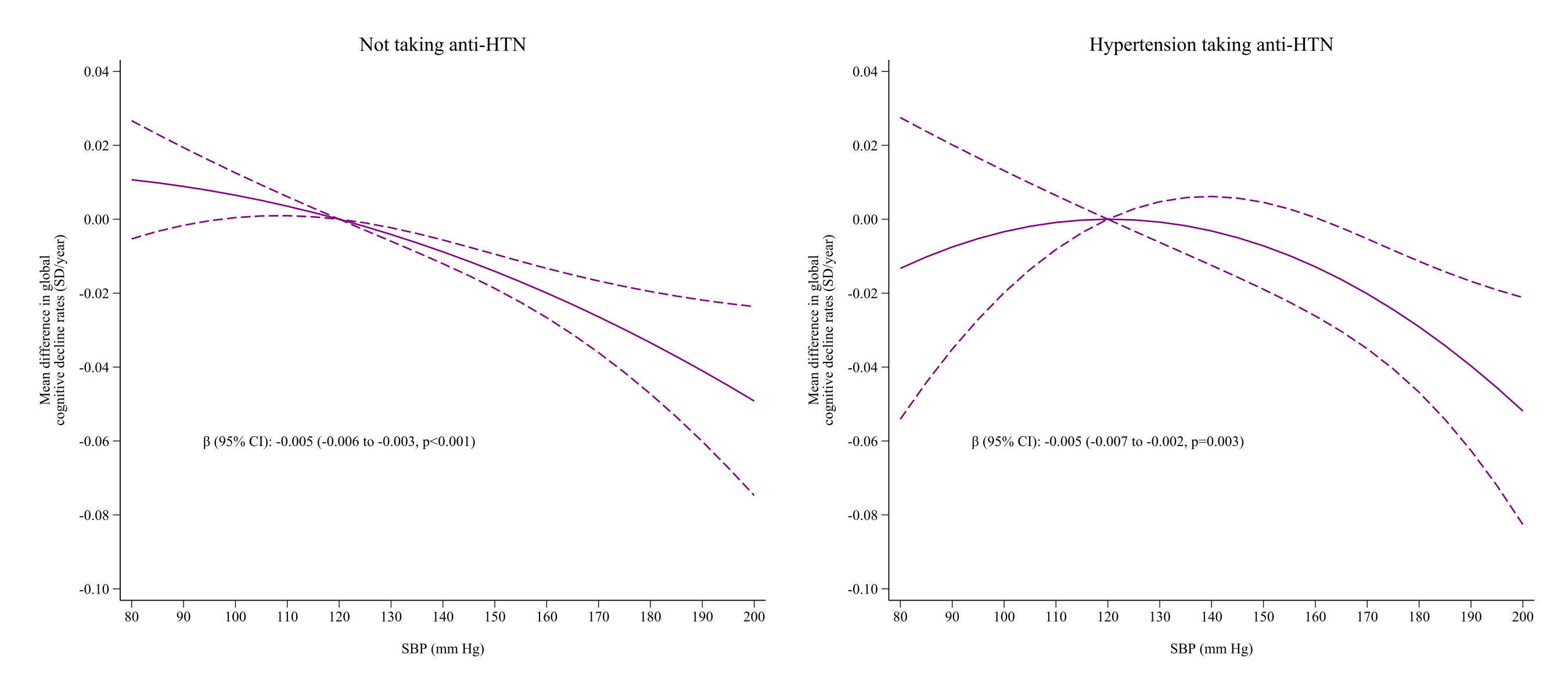


1. **PP and global cognitive Z scores by anti-HTN status**


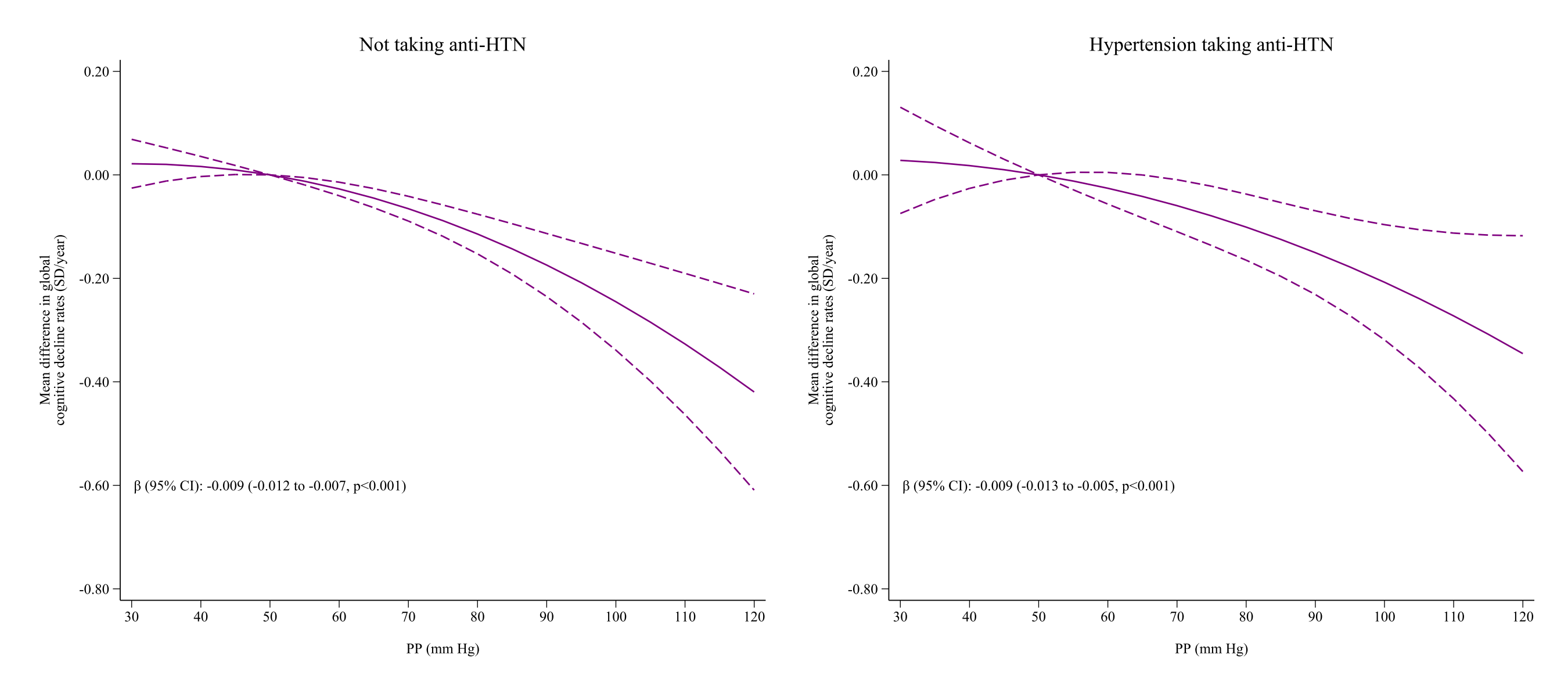


**Figure S6. Association Between SBP, PP and Rate of Global Cognitive Decline (SD/Year) Over 7 Years of Follow-Up, By Anti-HTN Status**

**Figure S7. Association Between SBP and Rate of Global Cognitive Decline (SD/Year) Over 7 Years of Follow-Up, with Additional Adjustment of Anti-Hypertensive Medication Use, Using <120 mmHg as the Reference Group**

Notes: *P* for trend < 0.001

**Figure S8. Association Between DBP and Rate of Global Cognitive Decline (SD/Year) Over 7 Years of Follow-Up, with Additional Adjustment of Anti-Hypertensive Medication Use, Using <60 mmHg as the Reference Group**

Notes: *P* for trend = 0.620

**Figure S9. Association Between PP and Rate of Global Cognitive Decline (SD/Year) Over 7 Years of Follow-Up, with Additional Adjustment of Anti-Hypertensive Medication Use, Using <40 mmHg as the Reference Group**

Notes: *P* for trend < 0.001

| **Table S1. Association Between Cumulative Blood Pressure Exposure and Global Cognitive Decline Rate (SD/Year) Over 5 Years of Follow-Up, Estimated by Linear Mixed Effects Regression, by Subgroup** | | | |
| --- | --- | --- | --- |
| Subgroup | Mean Difference (95% CI) in Rate of Change (SD/Year) | *P* value | *P* value for Interaction |
| Cumulative SBP, mm Hg × y, per SD increment | | | |
| Age group |  |  | 0.998 |
| <60 years | -0.009 (-0.017 to -0.001) | 0.02 |  |
| ≥60 years | -0.008 (-0.015 to -0.001) | 0.03 |  |
| Sex |  |  | 0.455 |
| Male | -0.013 (-0.023 to -0.004) | 0.005 |  |
| Female | -0.012 (-0.018 to -0.006) | <0.001 |  |
| Cumulative PP, mm Hg × y, per SD increment | | | |
| Age group |  |  | 0.274 |
| <60 years | -0.018 (-0.027 to -0.009) | <0.001 |  |
| ≥60 years | -0.008 (-0.015 to -0.002) | 0.016 |  |
| Sex |  |  | 0.193 |
| Male | -0.018 (-0.028 to -0.009) | <0.001 |  |
| Female | -0.017 (-0.023 to -0.011) | 0.016 |  |

| **Table S2. Association Between Cumulative Blood Pressure Exposure and Global Cognitive Decline Rate (SD/Year) Over 5 Years of Follow-Up, Estimated by Linear Mixed Effects Regression, Excluding Those with Cardiovascular Diseases, Diabetes, or Both at Baseline** | | |
| --- | --- | --- |
| Subgroup | Mean Difference (95% CI) in Rate of Change (SD/Year) | *P* value |
| Cumulative SBP, mm Hg × y, per SD increment | | |
| Excluding CVDs at baseline | -0.013 (-0.023 to -0.004) | <0.001 |
| Excluding diabetes at baseline | -0.010 (-0.015 to -0.005) | <0.001 |
| Excluding both at baseline | -0.011 (-0.017 to -0.005) | <0.001 |
| Cumulative PP, mm Hg × y, per SD increment | | |
| Excluding CVDs at baseline | -0.019 (-0.024 to -0.013) | 0.02 |
| Excluding diabetes at baseline | -0.016 (-0.021 to -0.011) | <0.001 |
| Excluding both at baseline | -0.017 (-0.023 to -0.012) | <0.001 |
